# Optimal Number of Message Transmissions for Probabilistic Guarantee of Latency in the IoT

**DOI:** 10.3390/s19183970

**Published:** 2019-09-14

**Authors:** Pascale Minet, Yasuyuki Tanaka

**Affiliations:** Inria Research Center of Paris, 75012 Paris, France

**Keywords:** IoT, industrial IoT, reliability, TSCH, latency, scheduling, network lifetime, IEEE802.15.4e, PDR, ETX, retransmission, probabilistic guarantee

## Abstract

The Internet of Things (IoT) is now experiencing its first phase of industrialization. Industrial companies are completing proofs of concept and many of them plan to invest in automation, flexibility and quality of production in their plants. Their use of a wireless network is conditioned upon its ability to meet three Key Performance Indicators (KPIs), namely a maximum acceptable end-to-end latency *L*, a targeted end-to-end reliability *R* and a minimum network lifetime *T*. The IoT network has to guarantee that at least R% of messages generated by sensor nodes are delivered to the sink with a latency ≤*L*, whereas the network lifetime is at least equal to *T*. In this paper, we show how to provide the targeted end-to-end reliability *R* by means of retransmissions to cope with the unreliability of wireless links. We present two methods to compute the maximum number of transmissions per message required to achieve *R*. MFair is very easy to compute, whereas MOpt minimizes the total number of transmissions necessary for a message to reach the sink. MFair and MOpt are then integrated into a TSCH network with a load-based scheduler to evaluate the three KPIs on a generic data-gathering application. We first consider a toy example with eight nodes where the maximum number of transmissions MaxTrans is tuned per link and per flow. Finally, a network of 50 nodes, representative of real network deployments, is evaluated assuming MaxTrans is fixed. For both TSCH networks, we show that MOpt provides a better reliability and a longer lifetime than MFair, which provides a shorter average end-to-end latency. MOpt provides more predictable end-to-end performances than Kausa, a KPI-aware, state-of-the-art scheduler.

## 1. Introduction

The Internet of Things (IoT) is transforming our daily life at home [1], at work, in our cities [2], in transportation [3], in sport training and healthcare, in process control and automation, in smart farming and beyond.

### 1.1. Context

At home or in the office, the IoT allows us to save time and energy by controlling lights and appliances and knowing our resource consumption habits. In business and industry, it increases productivity and efficiency by streamlining processes. In transportation, it helps people to enjoy services of better quality. IoT devices are electronic devices able to communicate with a network and perform a task. We can draw a distinction between consumer devices, smart home devices, enterprise and industrial IoT devices. In this paper, we do not focus on IoT devices themselves, but rather on applications using these devices. More precisely, we will consider IoT applications that are responsible for data gathering and are the most demanding in terms of quality of service provided by the networks supporting them [4,5]. We will consider three types of requirements:

- *end-to-end reliability*, denoted *R*: the data generated by any sensor node must be delivered to the sink with a probability greater than or equal to *R*. A value of 0.99 is frequent. In industry, a value of 0.99999 is targeted.
- *end-to-end latency*, denoted *L*: since, on the one hand, the role of the network is to collect data generated by wireless sensor nodes that are deployed in the area covered by this network and, on the other hand, only up-to-date information can be used to take accurate decisions, the time elapsed between data generation and data delivery to the sink is defined as the end-to-end latency. A value less than one second is usual.
- *network lifetime*, denoted *T*: since most IoT devices are battery operated, the goal of the network is to maximize its lifetime defined as the time when the first device has exhausted its battery. The importance of this requirement increases with the difficulty or the cost of replacing a battery in the environment concerned. A lifetime of several years (e.g., 3 years) is usual.

Since most wireless sensor networks deployed up to now are based on the IEEE 802.15.4 technology [6] and, given that the TSCH (Time Slotted Channel Hopping) technology [7] has been designed to enhance the IEEE 802.15.4 technology to better meet the three types of requirements previously defined, we focus our study on TSCH networks. Is a TSCH network able to meet the usual requirements of IoT applications? The goal of this paper is to answer this question, which can be refined into: is a TSCH network capable of ensuring that at least *R* percent of messages will be delivered to the sink with a delay less than or equal to *L*, and a network lifetime greater than or equal to *T*, while taking into account the unreliability of wireless links.

### 1.2. Contributions

Before going further, we define the concept of flow. A flow is defined as a set of messages having the same attributes such as the source address, the destination address and the QoS (Quality of Service) parameters (e.g., end-to-end reliability, end-to-end latency).

In contrast to many network performance evaluations, we take into account the unreliability of wireless links. The first contribution of this paper is to show how to reach the targeted end-to-end reliability *R* with two methods MFair and MOpt able to estimate the maximum number of transmissions per message and per flow over each link visited, taking into account both the heterogeneity of link quality and the dynamicity of link quality observed on real traces. Whereas MFair is very simple, MOpt minimizes the total number of transmissions per message, for each flow considered. The second contribution consists of providing a fully-integrated approach for a TSCH network by integrating these methods and a Load-based scheduler in the 6TiSCH stack. As a third contribution, we evaluate the performances (i.e., the end-to-end reliability and the end-to-end latency) obtained by this approach on two TSCH networks, one of eight nodes and the other of 50 nodes. Finally, we show that, on a generic data-gathering application, this approach provides more predictable end-to-end performances than Kausa [8], a KPI-aware state-of-the-art scheduler.

This paper is organized as follows. In Section 2, we present the advantages and drawbacks of well-known link quality estimators and show how they are used to enhance network performances. Since the approach proposed in this paper shares the same goals as Kausa [8], Kausa is briefly described. A more detailed description is given in Section 7. Section 3 describes the two methods we propose to estimate the maximum number of transmissions per message and per flow over each link visited. Section 4 shows how to integrate these methods in a TSCH network with a centralized scheduling function. Section 5 gives performance results with regard to end-to-end reliability and end-to-end latency for a targeted end-to-end reliability ranging from 0.9 to 0.99999 for a toy example. In Section 6, a TSCH network of 50 nodes is considered with a generic data-gathering application. End-to-end latency and reliability are evaluated. The solution proposed is compared to Kausa in Section 7. Finally, we conclude in Section 8.

## 2. Related Work

Different ways to estimate the quality of a wireless link exist. They all rely on link measurements obtained by an active, passive or hybrid link monitoring. A usual classification distinguishes between hardware and software-based link quality estimators [9,10]. Hardware estimators have the advantage of being directly provided by hardware without the need for any additional processing overhead. However, their accuracy is not very good for two reasons. First, they are measured only on successfully received packets, and they do not take into account the number of packet losses. Second, they are not evaluated on the whole packet received but only on eight symbols of this packet. The main hardware-based quality estimators are RSSI (Received Signal Strength Indicator), LQI (Link Quality Indicator), and SNR (Signal-to-Noise Ratio). In addition, LQI depends on the manufacturer of the radio transceiver used. For the software-based quality estimators, we distinguish those based on PRR (Packet Reception Rate), from those based on RNP (Required Number of Packet retransmissions) and finally those using a score. For instance, ETX (Expected Transmission Count) [11] is classified as an RNP-based estimator, whereas F-LQE (Fuzzy Link Quality Estimator) [12] is a score-based one assessing link quality in terms of four properties: Smoothed Packet reception Ratio, Stability factor, ASymmetry Level and channel Average Signal-to-Noise Ratio. In [13], the authors introduce Bmax, the maximum count of consecutive transmissions needed for a frame to be transmitted on a link. This metric evaluates the link burstiness, where a burst is defined as a period of continuous packet loss. It is obtained empirically for each link based on previous transmissions and can be seen as an observed worst case. Then, they build a schedule that allocates Bmax cells for every frame that a link is supposed to carry. The problem with such a method is that it may require a long delay (more than 140 hours according to the authors) before a representative Bmax is known for all the links.

Whatever the link quality estimator used, authors usually agree on the following conclusions:

- *The quality of any wireless link can be classified as good, intermediate or bad*. The challenge lies in an accurate estimation of the intermediate link quality.
- *An ideal link quality estimator should be* [10] *energy efficient* (i.e., requiring low processing, communication and memory overhead), *accurate* (i.e., reflecting the real link behavior), *reactive* (i.e., able to promptly react to persistent link state changes) *and stable* (i.e., able to tolerate transient link state changes).
- *To better reflect the real behavior of a link, several link properties should be taken into account*. That is why link quality estimators tend to combine several simple estimators [14] and use sophisticated techniques (e.g., simple average, filtering, machine learning [15,16], regression, and fuzzy logic [17,18]) to produce a metric from link measurements.

Link quality estimators have been used to improve the quality of service provided to end-users [19]. By using links of better quality, the network throughput is maximized, the delivery times are minimized, routes are more stable [18], etc. These improvements can be increased by link quality prediction using online machine learning techniques as in [20]. In such a case, packets are then able to avoid links before their quality degrades below an acceptable threshold, and routing reactivity is improved. Furthermore, if both the link quality estimator and the routing protocol take energy into account, network lifetime is maximized, as in [21].

Closer to our work, Gaillard et al. propose a greedy algorithm that optimizes the distribution of links used by flows network-wide [22]. These authors extend TASA [23], a well-known centralized scheduling algorithm for FTDMA networks, to take into account retransmissions and fragmented packets. They build a schedule that complies with reliability expectation by adding extra cells that are used in the case of consecutive retransmissions. They take into account link quality and packet fragmentation. The same authors further extend their research in [8] by proposing Kausa, a centralized scheduling algorithm that builds resource paths that guarantee QoS per flow, when multiple applications are using the same network, and each application has its own requirements and traffic flows.

In this paper, we propose to use a simple link quality estimator: *PDR (Packet Delivery Rate) that is easily computed by the sender as the ratio of the number of acknowledged frames to the number of sent frames*. Even if this estimator is not ideal, as previously defined, it provides an accurate estimation of link behavior because, unlike hardware-based link estimators, it takes into account packet losses. We use the PDR link quality estimator to compute the maximum number of transmissions per message and per flow over any link visited by the flow considered.

We share the same goal as Kausa [8]: namely, to build a centralized schedule ensuring that flows meet their required end-to-end latency and delivery rate, by means of message retransmissions. Like [8], we adopt a per flow approach, allowing us to differentiate QoS per flow. However, unlike [8], we consider the optimal solution for any flow to be the one that minimizes the total number of transmissions per message of this flow, while achieving the desired end-to-end reliability. Furthermore, traces obtained from real IoT networks like [24,25] allow us to better understand the challenges of wireless networking and make realistic assumptions. That is why, in this paper, the performance evaluation is based on PDR values computed from a realistic model, as explained in Section 6.1.

## 3. Optimal Retransmission Estimation

To cope with the unreliability of network links, unacknowledged messages are retransmitted up to a maximum number of retransmissions. We have to determine this maximum number that of course depends on the unreliability of the link considered as well as the targeted end-to-end reliability *R*.

Since each flow may request its own QoS, we adopt a per-flow approach. Without loss of generality, we assume that each message is labeled with its flow tag, which includes the origin node of the flow.

### 3.1. Assumptions and Basic Properties

Let us consider the flow *f* originating from any sensor node $N_{h}$ with h∈[1,n]. Let *h* denote the path length (i.e., the number of hops) from $N_{h}$ to the sink $N_{0}$. To simplify the notation, we assume that the path is defined by $N_{h}\to N_{h-1}\to N_{h-2}\cdots \to N_{0}$.

For any link j∈[1,h] of *f*’s path, we denote by $M_{j}$ the maximum number of transmissions of any message of *f* transmitted by node $N_{j}$ to its parent in the routing tree, and $P_{j}$ the probability of successful acknowledgment receipt after a single transmission, whereas $R_{j}$ is the probability of successful acknowledgment receipt on link *j* after a maximum number $M_{j}\geq 1$ of transmissions. Table 1 gives the notations used in this paper.

For the sake of simplicity, we adopt the following assumptions:

**Assumption** **1.** *Network links fail independently.*

**Assumption** **2.** *The transmissions of any message fail independently.*

**Assumption** **3.** *Each node has enough Transmission opportunities to prevent message drops due to Transmission queue overflow.*

In a TSCH network, this assumption means that the scheduling algorithm has assigned enough transmission cells to each node. As a consequence, the unreliability of a transmission is only due to the unreliability of the wireless link considered. If this assumption were not true, then the computation of the transmission reliability should include not only the message loss due to the unreliability of the wireless link considered but also the message loss due to Transmission queue overflow.

We first recall some basic properties:

**Property** **1.** *The end-to-end reliability on a path is equal to the product of the reliability of each link composing that path.*

**Property** **2.** *The probability of successful acknowledgment receipt over any link j increases with the maximum number of message transmissions $M_{j}$ according to the following equation:*

(1) $$R_{j}=1-{\left(1-P_{j}\right)}^{M_{j}}.$$

**Proof.**  After $M_{j}$ transmissions, the acknowledgment of a message is not received successfully with a probability equal to ${\left(1-P_{j}\right)}^{M_{j}}$. Hence, the probability of successful acknowledgment receipt after $M_{j}$ transmissions is equal to $1-{\left(1-P_{j}\right)}^{M_{j}}.$ □

### 3.2. A Fair Method

The question is: knowing the end-to-end reliability that must be met on a given path, how to distribute this targeted end-to-end reliability into the targeted reliability of each link on that path? The maximum number of transmissions on each link is then deduced from the targeted reliability on the link and the probability of successful transmission on that link. In this paper, we propose two methods that can be applied to any network meeting Assumptions 1–3.

Since we adopt a per-flow approach, we consider any flow *f*, originating from a node *h* hops away from the sink. In other words, the path of *f* consists of *h* links, h≥1. Since the end-to-end reliability on the path is equal to the product of the reliability of each of its links, which should be ≥R, a simple solution consists of fairly and uniformly sharing the end-to-end reliability over each link of the path. Hence, each link *j* has to meet a reliability equal to $R_{j}=R^{1/h}$. The maximum number of transmissions on any link *j* is then equal to $M_{j}=\left\lceil \frac{Log(1-R^{1/h})}{Log(1-P_{j})}\right\rceil$. This principle is applied by the MFair Algorithm given hereafter (see Algorithm 1).

**Algorithm 1:**MFair**:** compute the number of transmissions on each link *j* to reach a reliability $R^{1/h}$ over this link per message of flow *f* visiting *h* links.**Require:***R* the targeted end-to-end reliability, $P_{j}$ = the success probability of receiving the  acknowledgment after a single message transmission over link *j* with 1≤j≤h **Ensure:**
$M_{j}$ is the minimum number of transmissions on link *j* to achieve $R^{1/h}$ **for** each link *j*
**do**  **if**
$P_{j}=1$
**then**   $M_{j}\leftarrow 1$  **else**   $M_{j}\leftarrow \left\lceil \frac{Log(1-R^{1/h})}{Log(1-P_{j})}\right\rceil$  **end if** **end for**

**Property** **3.** *The processing complexity of the MFair Algorithm is in O(1).*

**Proof.**  The complexity of the Fair Algorithm is equal to the complexity of computing $\left\lceil \frac{Log(1-R^{1/h})}{Log(1-P_{j})}\right\rceil$ for each link, times *h* the number of links, hence the property. □

**Figure 1:**
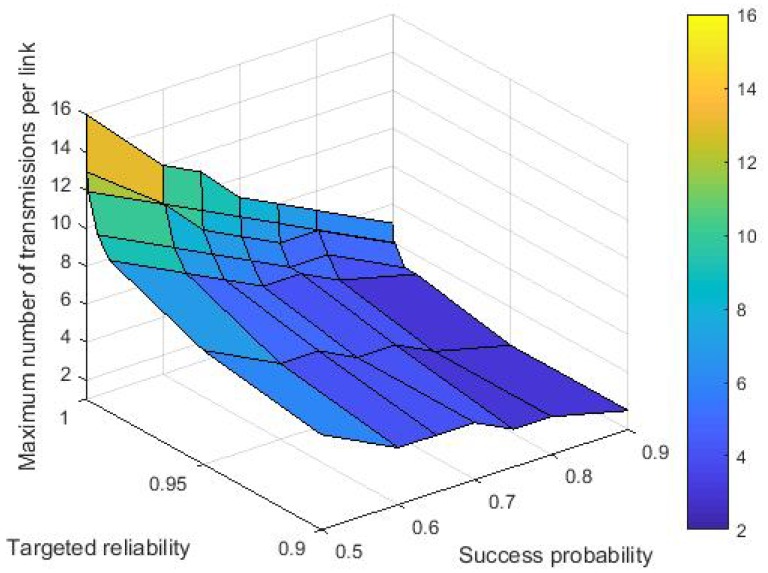
Maximum number of transmissions over a link visited by a 4-hop flow, computed by MFair.

Figure 1 depicts the maximum number of transmissions over a link whose probability of successful acknowledgment receipt ranges from 0.5 to 0.9 for a 4-hop flow when the targeted end-to-end reliability ranges from 0.9 to 0.9999.

### 3.3. An Optimal Method

For any sensor node $N_{i}$, let us denote *f* the flow originating from $N_{i}$. The problem consists of minimizing the total number of transmissions needed to deliver any message of *f* to the sink, under the constraint that the end-to-end latency is greater than or equal to *R*.

The optimization problem can be defined as follows:

**Goal: Find the values of $M_{j}$ for each link *j* visited by *f*, j=1⋯h that minimize $\sum_{j=1}^{h}M_{j}$ under the constraints:**

(2) $$\prod_{j=1}^{h}\left(1-{\left(1-P_{j}\right)}^{M_{j}}\right)\geq R,$$

(3) $$M_{j}\;\mathrm{integer}\geq 1.$$

If several solutions exist for the same total number of transmissions, choose the solution maximizing $\prod_{j=1}^{h}\left(1-{\left(1-P_{j}\right)}^{M_{j}}\right)$.

**Lemma** **1.** *Each link j=1⋯h should provide a reliability $R_{j}$ at least equal to the requested end-to-end reliability R, $R_{j}\geq R$. Hence, $M_{j}\geq \left\lceil \frac{Log(1-R)}{Log(1-P_{j})}\right\rceil .$*

**Proof.**  If there is a link *j* that provides a reliability $R_{j}<R$, then the product of the reliability of all other links should be greater than 1 to obtain an end-to-end reliability of *R*, which is impossible. Therefore, each link has to provide a reliability at least equal to *R* by means of retransmissions. Since the reliability of a link *j* increases with the maximum number of transmissions according to Equation (1), to obtain an end-to-end reliability of *R*, the number of transmissions $M_{j}$ should be greater than or equal to that needed to obtain a link reliability of *R*, leading to $M_{j}\geq \left\lceil \frac{Log(1-R)}{Log(1-P_{j})}\right\rceil .$ □

**Lemma** **2.** *There is no solution ensuring an end-to-end reliability ≥R, with a total number of transmissions $<\sum_{j=1}^{h}\left\lceil \frac{Log(1-R)}{Log(1-P_{j})}\right\rceil .$*

**Proof.**  It is deduced from Lemma 1. □

**Lemma** **3.** *If there is no solution ensuring an end-to-end reliability greater than or equal to R, with a total number of transmissions $\leq M=\sum_{j=1}^{h}\left\lceil \frac{Log(1-R)}{Log(1-P_{j})}\right\rceil$ and, if increasing $M_{j}$ to $M_{j}+1$ for the link j maximizing the value of $P_{j}\left(1/R_{j}-1\right)$ does not achieve R, then there is no solution ensuring an end-to-end reliability R in M+1 transmissions.*

**Proof.**  Assuming that there is no solution ensuring an end-to-end reliability ≥R, with a total number of transmissions $\leq M=\sum_{j=1}^{h}\left\lceil \frac{Log(1-R)}{Log(1-P_{j})}\right\rceil$, we compute, for any link *k*, the benefit on the end-to-end reliability brought by increasing $M_{k}$ to $M_{k}+1$. The new end-to-end reliability can be written $newR\;=\;\left(\prod_{j=1,j\neq k}^{h}R_{j}\right)\left(1-{\left(1-P_{k}\right)}^{M_{k}+1}\right)$. Since $1-{\left(1-P_{k}\right)}^{M_{k}+1}=1-\left(1-P_{k}\right){\left(1-P_{k}\right)}^{M_{k}}=R_{k}+P_{k}\left(1-R_{k}\right)$, we get $newR=\left(\prod_{j=1,j\neq k}^{h}R_{j}\right)\left(R_{k}+P_{k}\left(1-R_{k}\right)\right).$ Denoting $\prod_{j=1}^{h}R_{j}$ as oldR, we get $newR=oldR(1-P_{k}+P_{k}/R_{k})$. Hence, maximizing newR means selecting the link *k* that maximizes $P_{k}\left(1/R_{k}-1\right).$ Hence, if with that link *k*, the end-to-end reliability newR is not greater than or equal to *R*, then no other link can meet the end-to-end reliability *R* with a total number of transmissions equal to M+1. □

**Property** **4.** *The MOpt algorithm given in Algorithm 2 finds the optimal solution.*

**Proof.**  The algorithm starts with the smallest possible number of transmissions *M*, according to Lemma 2. If the requested end-to-end reliability is met, then the solution is found. Otherwise, the algorithm increases the total number of transmissions by one and checks again whether the requested end-to-end reliability is met by increasing the number of transmissions of one on the link providing the highest reliability gain. If yes, the solution is found, otherwise there is no solution for M+1 transmissions. □

**Algorithm 2:**MOpt**:** Compute the number of transmissions $M_{j}$ on link *j* to achieve an end-to end reliability ≥R and minimize the total number of transmissions per message of flow *f*
visiting *h* links**Require:***R* the targeted end-to-end reliability 0<R<1, $P_{j}$=the probability of successful  acknowledgment receipt after a single transmission over link *j* with 1≤j≤h **Ensure:**
$M_{j}$ is the minimum number of transmissions to achieve *R* **for** each link *j*
**do**  **if**
$P_{j}=1$
**then**   $M_{j}\leftarrow 1$  **else**   $M_{j}\leftarrow \left\lceil \frac{Log(1-R)}{Log(1-P_{j})}\right\rceil$   $R_{j}\leftarrow 1-{\left(1-P_{j}\right)}^{M_{j}}$   $Gain_{j}\leftarrow P_{j}\left(1/R_{j}-1\right)$  **end if** **end for** **while**
$\prod_{j=1}^{h}R_{j}<R$
**do**  Select the link *j* maximizing $Gain_{j}$  If several links provide the same Gain, take the farthest link *j* from the sink  $M_{j}\leftarrow M_{j}+1$  $R_{j}\leftarrow 1-{\left(1-P_{j}\right)}^{M_{j}}$  $Gain_{j}\leftarrow P_{j}\left(1/R_{j}-1\right)$ **end while**

We now evaluate the complexity of this algorithm and more particularly we upper bound the number of iterations needed to find the maximum number of transmissions over each link such that the total number of transmissions per message of the flow considered is minimized.

**Property** **5.** *The MOpt Algorithm, given in Algorithm 2, finds the optimal solution in a number of iterations less than or equal to $\sum_{j=1}^{h}\left\lfloor \frac{Log(h)}{Log(1-P_{j})}\right\rfloor$.*

**Proof.**  Let us consider any link *j* visited by the flow considered. Since R→1, we have $R^{1/h}\;<\;1\;-\;\frac{1-R}{h}$. Hence, $Log\left(1-R^{1/h}\right)>Log\left(1-R\right)-Log\left(h\right)$, leading to: $\frac{Log(1-R^{1/h})}{Log(1-P_{j})}\;<\;\frac{Log(1-R)-Log(h)}{Log(1-P_{j})}$. We then obtain $\left\lceil \frac{Log(1-R^{1/h})}{Log(1-P_{j})}\right\rceil \leq \left\lceil \frac{Log(1-R)-Log(h)}{Log(1-P_{j})}\right\rceil$. Since ⌈a−b⌉≤⌈a⌉−⌊b⌋, we get: $M_{j,fair}\;-\;M_{j,init}\;\leq \;\left\lfloor \frac{Log(h)}{Log(1-P_{j})}\right\rfloor$, where $M_{j,fair}$ denotes the maximum number of transmissions over link *j*, whereas $M_{j,init}$ denotes the first value tried by the optimal algorithm. Since $\sum_{j=1}^{h}M_{j,fair}$ is the maximum number of transmissions of a message to reach the sink provided by the Fair algorithm and this number is never exceeded by the Optimal algorithm, the maximum number of iterations of the Optimal algorithm is upper bounded by $\sum_{j=1}^{h}M_{j,fair}-\sum_{j=1}^{h}M_{j,init}\leq \left\lfloor \frac{Log(h)}{Log(1-P_{j})}\right\rfloor$, hence the property. □

### 3.4. Expected vs. Maximum Number of Transmissions

For any flow considered and whatever the method adopted to compute the maximum number of transmissions over each link visited by the flow considered, the real number of transmissions used is much smaller than the maximum one, which occurs with a very low probability. In this section, we want to evaluate the energy saving obtained with a variable number of transmissions instead of always considering the worst case that has a very low occurrence probability. With the Assumptions 1–3, we can evaluate $E(M_{j})$ the expected number of transmissions on any link *j*, knowing that $M_{j}$, the maximum number of transmissions, is computed either by MFair or by MOpt. We then have:

$E\left(M_{j}\right)=\sum_{k=1}^{M_{j}-1}kP_{j}{\left(1-P_{j}\right)}^{k-1}+M_{j}{\left(1-P_{j}\right)}^{M_{j}-1}$.

**Property** **6.** *The expected number of transmissions on any link j is equal to*

$$E\left(M_{j}\right)=\frac{1-{\left(1-P_{j}\right)}^{M_{j}}}{P_{j}},$$

*where $M_{j}$ denotes the maximum number of transmissions used for link j.*

**Proof.**  Let us consider $S_{n}\left(x\right)=\sum_{k=1}^{n}{\left(1+x\right)}^{k}$ and let $S_{n}^{\prime }\left(x\right)$ be its derivative. We have $S_{n}^{\prime }\left(x\right)=\sum_{k=1}^{n}k{\left(1+x\right)}^{k-1}$. Since $S_{n}\left(x\right)$ is the sum of a geometric progression, we have:

$S_{n}\left(x\right)=-\frac{1+x-{\left(1+x\right)}^{n+1}}{x}=\frac{\left(1+x\right)({\left(1+x\right)}^{n}-1)}{x}$.

Hence, the derivative is:

$S_{n}^{\prime }\left(x\right)=\frac{{\left(1+x\right)}^{n}\left(nx-1\right)+1}{x^{2}}$. By replacing *x* by $-P_{j}$ and *n* by $M_{j}-1$, we get

$E\left(M_{j}\right)=\sum_{k=1}^{M_{j}-1}kP_{j}{\left(1-P_{j}\right)}^{k-1}+M_{j}{\left(1-P_{j}\right)}^{M_{j}-1}=\frac{1-{\left(1-P_{j}\right)}^{M_{j}-1}\left(-1-\left(M_{j}-1\right)P_{j}\right)+1}{P_{j}}=\frac{1-{\left(1-P_{j}\right)}^{M_{j}}}{P_{j}}$, hence the property. □

**Figure 2:**
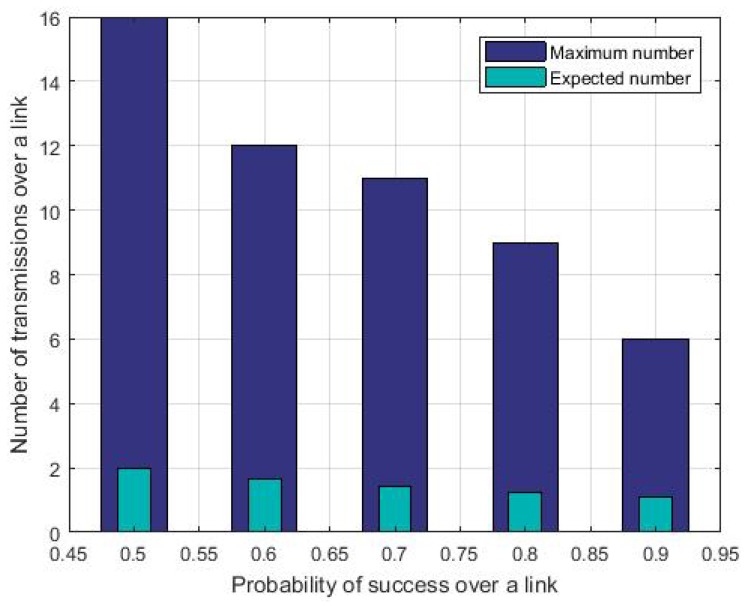
Expected number of transmissions versus MFair Maximum number of transmissions over a link.

Figure 2 depicts the maximum and the expected numbers of transmissions per message of a 4-hop flow, for a targeted end-to-end reliability of 0.9999, over a link whose success probability per transmission ranges from 0.5 to 0.9. We observed that, in all the cases studied, although the value of $E(M_{j})$ decreases when $P_{j}$, the success probability per transmission increases, $\left\lceil E\left(M_{j}\right)\right\rceil$ remains constant and equal to 2. By stopping its retransmissions as soon it receives an acknowledgment, the sender saves its energy. For instance, let us consider that for any link *j* for which $P_{j}=0.5$ is visited by a 4-hop flow, the sender saves $M_{j}-\left\lceil E\left(M_{j}\right)\right\rceil$ transmissions for this link that is 16−2=14 transmissions for MFair. Since the flow visits four hops and assuming that its four links have the same success probability, each of the four transmitting nodes saves 14 transmissions. Assuming that the schedule is periodic with a period of 101 time slots (the default value in the TSCH network), and this flow generates a message per schedule period, these four nodes save 140 ms each schedule period of 101×7.25=732.25 ms, for a slot duration of 7.25 ms. This corresponds to an increase of 19% in sleeping time per schedule period for each node.

It is useful to compare this value with the value of ETX on link *j*, denoted by $ETX_{j}$. Let $D_{j}$ be the delivery rate from node *j* to its parent, whereas $D_{r(j)}$ is the delivery rate in the reverse direction. Since $P_{j}$ is defined as the probability for *j* to receive the acknowledgment of its message, we have to take into account the delivery rates of both directions, which gives:

(4) $$P_{j}=D_{j}*D_{r(j)}$$

$ETX_{j}$ is defined as:

(5) $$ETX_{j}=\frac{1}{D_{j}*D_{r(j)}}.$$

As a consequence, $ETX_{j}$ does not depend on the targeted end-to-end reliability *R* but only on the reliability of both directions of the link considered. This explains the main difference between $ETX_{j}$ and $M_{j}$.

In addition, $ETX_{j}$ is not equal to E(j). Indeed, E(j) assumes a maximum number of transmissions equal to $M_{j}$, whereas $ETX_{j}$ assumes a number of transmissions that may be infinite.

## 4. Framework for a TSCH Network

The MOpt and MFair methods are now applied to compute the maximum number of transmissions per link and per flow. Flows are generated by a low-power network based on the TSCH technology [7].

We focus on data gathering applications with end-to-end requirements in terms of reliability and latency, as well as requirements with regard to network lifetime. The network supporting these applications is a TSCH network [7].

### 4.1. TSCH Network

In a TSCH network, the medium access is time-slotted and several transmissions are done on different channels in the same time slot. More precisely, transmissions are scheduled in cells, where a cell is defined by its channel offset and its time slot offset. There are two types of cells: shared cells where any node having a message to transmit is allowed to do so, and dedicated cells, where only the transmitter defined in the schedule is allowed to. The choice of a wireless TSCH network helps to meet Assumptions 1–3 because the mapping between logical channels and physical ones changes at each time slot. Thus, even if a message is retransmitted in the next slot and on the same logical channel as previously, it will be transmitted on a different physical channel.

The schedule of transmissions is periodic and conflicts in dedicated cells are avoided. In addition, nodes know from the schedule in which slots they are allowed to transmit or to receive. They sleep in any other slot in order to save energy.

### 4.2. Scheduling Function

The scheduling algorithm, which is assumed to be centralized in this paper, works per flow: it allocates the cells needed to transmit a message from the flow origin to the sink. More precisely, it proceeds hop by hop, starting from the flow origin and allocating to each visited node the number of cells needed to receive the message from its child and then the number of cells needed to transmit this message to its parent. Since the scheduler does not know a priori which message transmission will be successful, it has to take into account the worst case where a message is received by the next hop after the maximum number of transmissions for this link and this flow. Hence, for each message, the scheduler allocates to each visited link a number of cells corresponding to the maximum number of transmissions on that link for the flow in question.

However, this does not mean that each message is transmitted a maximum number of times. In fact, as soon as the sender has received the acknowledgment of any message msg, it stops retransmitting msg and may use the slot foreseen for a retransmission of msg for the transmission of another message, if it has one in its Transmit queue.

Any sensor node first transmits the message in its Transmit queue that has the highest flow priority, as the primary criterion and the smallest timestamp within a same flow, as the secondary criterion. This assumes that messages are timestamped when they are generated by their origin node.

The Load-based scheduler is selected because of its simplicity combined with its very good performances [26]. This scheduler schedules first the flow originating from the most loaded node. The load of a node is computed as the number of cells needed to transmit its own flows, plus the number of cells needed to receive and transmit the flows originating from its descendants.

### 4.3. Computation of Key Performance Indicators

In this paper, we consider three Key Performance Indicators (KPIs) that matter for Industry 4.0 and the IoT. We now show how to compute them for a TSCH network and a scheduling function defined in Section 4.1 and Section 4.2, respectively. These three KPIs are:

- *The maximum end-to-end latency L* is the maximum time elapsed between data generation by a sensor node and its delivery to the sink. To compute this value within the framework defined in Section 4.2, we make an additional assumption:
  **Assumption** **4.** The maximum number of message transmissions on a link, denoted as MaxTrans, a parameter of the MAC TSCH protocol, is dynamically tuned according to the value computed by MFair or MOpt.
  With Assumption 4, the maximum end-to-end latency [27] is obtained when the last slot assigned to the node considered has just elapsed and then only the last transmission of the message is successful. This gives:
  (6) (SlotframeSize−1+UsedSlots)×SlotDuration,
  where UsedSlots is the number of slots used by the schedule for data gathering. Hence, the smallest maximum end-to-end latency that can be achieved is obtained by an optimal schedule, which uses MinSize the minimum number of slots for data gathering and for a slotframe duration equal to this number of slots. This smallest maximum latency is equal to
  (7) (2*MinSize−1)×SlotDuration.
- *The end-to-end reliability R* provided by the network. It is evaluated by the ratio of the total number of user-data messages sent by the sensor nodes over the total number of user-data messages delivered to the sink.
- *Network lifetime T* is defined as the time the first node runs out of battery. Network lifetime can be expressed as:
  (8) $$\underset{N\in Nodes}{Min}\;\frac{Initial\_Energy(N)\times SFDuration}{Average\_Energy\_Consumption(N)},$$
  where Initial_Energy(N) denotes the initial energy of node *N*, Average_Energy_Consumption(N) is the average energy consumption of *N* per slotframe, and SFDuration is the slotframe duration.

To evaluate the network lifetime, defined as the time up to first battery depletion of the busiest node, we use the parameters whose values are given in Table 2.

To summarize, the IoT network has to guarantee that at least *R* percent of the messages generated by sensor nodes are delivered to the sink with a latency ≤L, whereas the network lifetime is at least equal to *T*.

### 4.4. Generalization of the Theoretical Bound on the Maximum Latency

We now compute a theoretical bound on the maximum latency when $TXCell_{f}\left(N_{i}\right)<MaxTrans$, where $TXCell_{f}\left(N_{i}\right)$ denotes the number of TX cells assigned to flow *f* on node $N_{i}$.

Let *f* be the flow whose message *m* has the maximum end-to-end latency. Let $N_{k}$ be the source node of *f* which is *k* hops away from the sink. Flow *f* visits successively $N_{k},N_{k-1},\cdots N_{1}$ and then the sink. We adopt an additional assumption: on any visited node, *m* is never delayed by another flow. The worst case occurs when on $N_{k}$ message *m* is generated just after the last slot assigned to $N_{k}$. Hence, $N_{k}$ has to wait for the next slotframe to transmit *m*. In addition, on any node, only the last transmission (i.e., the $MaxTrans^{th}$ transmission) is received in the worst case; the previous ones are lost. According to the framework defined in Section 4.2, when any node $N_{i}$ receives a message of flow *f* in a slotframe, it has $TXCell_{f}\left(N_{i}\right)$ cells to transmit it to its parent in the current slotframe. In each slotframe, any node $N_{i}$ has $\sum_{g}TXCell_{g}\left(N_{i}\right)$ opportunities to transmit a message to its parent, where *g* is a flow visiting $N_{i}$. Hence, we get the following formula:

(9) $$MaxLatency\left(f\right)\leq \left(\left(\left\lceil \frac{MaxTrans}{TXCell_{f}\left(N_{k}\right)}\right\rceil +\sum_{h=1}^{k-1}\left\lfloor \frac{MaxTrans-TXCell_{f}\left(N_{h}\right)}{\sum_{g}TXCell_{g}\left(N_{h}\right)}\right\rfloor \right)*SlotframeSize+SlotUsed\right)*SlotDuration.$$

If only Depth, the routing tree depth, and MinTXCell, the minimum number of TX cells per pair (sensor node, flow), are known, the bound becomes, taking into account that each sensor node generates its own flow:

(10) $$MaxLatency\leq \left(\left(\left\lceil \frac{MaxTrans}{MinTXCell}\right\rceil +\sum_{h=1}^{k-1}\left\lfloor \frac{MaxTrans-MinTXCell}{MinTXCell*(Depth-h+1)}\right\rfloor \right)*SlotframeSize+SlotUsed\right)*SlotDuration.$$

Notice that Equations (9) and (10) generalize Equation (6), which is valid only when MinTXCell≥MaxTrans.

## 5. Performance Results for a Toy Example

**Figure 3:**
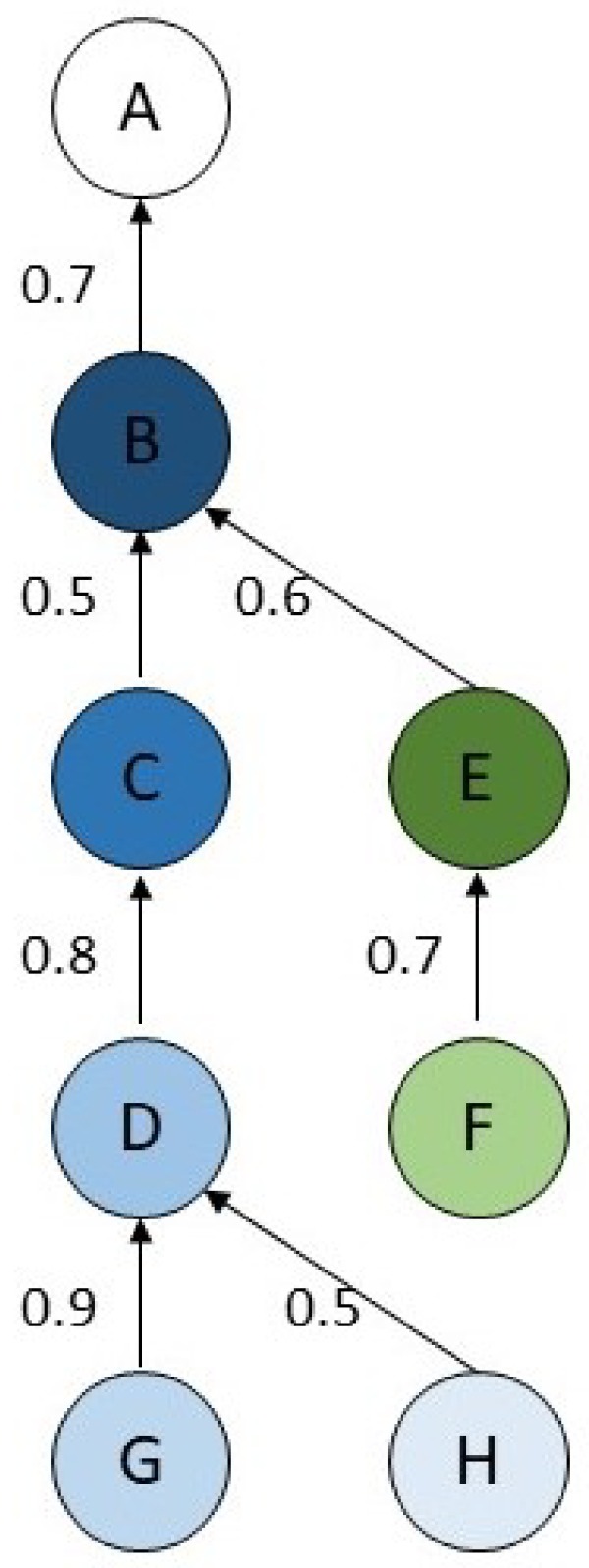
A routing tree with the PDR of each link used by flows.

We first consider a toy example of a wireless TSCH network comprising a sink and seven sensor nodes. Each sensor node generates an application message of 27 bytes every 10 s. The slot duration is assumed to be 7.25 ms. The routing tree is depicted in Figure 3, where node *A* denotes the sink. The value associated with each link *j* gives $P_{j}$ the probability of successful receipt of a single transmission over that link. We notice that links have heterogeneous qualities, ranging from 0.5 to 0.9. For each of the seven flows generated by a sensor node, we compute the maximum number of transmissions per link for any message of this flow. All the flows, except that generated by *B*, are multi-hop, which is six flows. In this example, we assume that Assumption 4 is met: MaxTrans is dynamically tuned according to the value computed by MFair or Mopt.

### 5.1. Number of Transmissions and End-To-End Reliability

**Figure 4:**
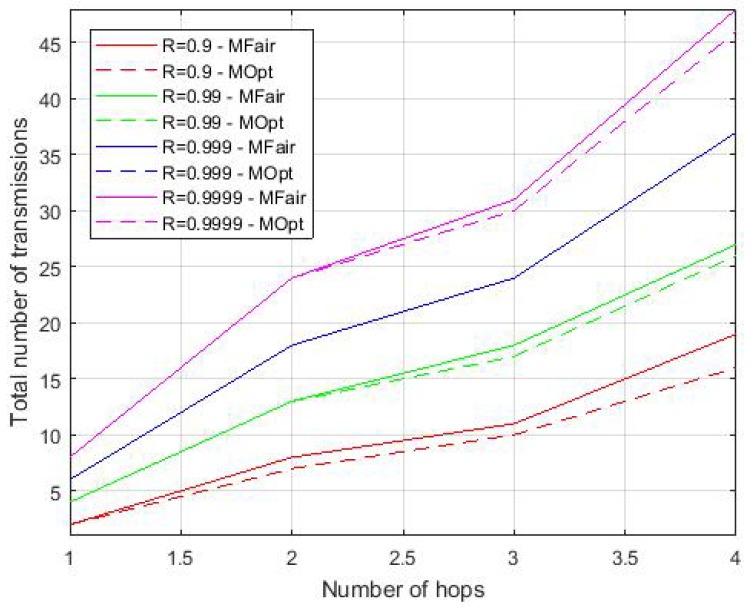
Total number of transmissions for a flow message.

Figure 4 depicts the total maximum number of transmissions for a message of a flow, depending on the hop number, when the targeted end-to-end reliability ranges from 0.9 to 0.9999. In this figure, the one-hop flow corresponds to the flow originating from *B*, whereas the two-hop flows correspond to the flows originating from *C* and *E*, the three-hop flows are those generated by *D* and *F* and the four-hop flows are those originating from *G* and *H*.

Table 3, Table 4, Table 5, Table 6 and Table 7 compare the values obtained with MFair and MOpt, when the targeted end-to-end reliability ranges from 0.9 to 0.99999. These tables show that, whatever the targeted reliability, and as expected:

- The maximum number of transmissions per message over a link increases, when the link quality decreases.
- Both methods provide the same maximum number of transmissions for all single-hop flows.
- The total number of transmissions per message of any given multi-hop flow obtained by MOpt is always less than or equal to that obtained by MFair. For instance, for R=0.9 (see Table 3), we observe a gain on the total number of transmissions per message and per flow, which is equal to 1 for the 2-hop flows (i.e., flows originating from *C* and *E*), and for the 3-hop flow originating from *D*. This gain becomes 2 for the 4-hop flow originating from *G* and 3 for the 4-hop flow originating from *H*. To summarize the results obtained for the six multi-hop flows considered, we observe five improvements for R=0.9, four improvements for R=0.99, two improvements for R=0.999, four improvements for R=0.9999 and five improvements for R=0.99999, leading to a total of 20 improvements over the 30 cases tested.
- Even if the total number of transmissions is the same for both methods, the distribution over the links may differ as exemplified in Table 7 by the flow originating from *D*, where MOpt gets an end-to-end reliability of 0.99922, whereas MFair gets a slightly less value 0.99921. Hence, MOpt provides the smallest total number of transmissions per flow and, if equal with MFair, MOpt provides the highest end-to-end reliability.
- With MFair, two links having the same link quality always have the same maximum number of transmissions for the same flow, as exemplified in all tables by the flow originating from *H*, where the links HD and CB have the same quality. However, this is not always the case with MOpt; see, for instance, this flow in Table 4, where the maximum transmission number for link HD is 9, whereas it is 8 for link CB. Since the nodes close to the sink usually have a larger load, decreasing their load improves the network performances. Notice, however, that the maximum number of transmissions on a given link depends on the flow. For instance, the maximum number of transmissions on Link B→A is equal to 11 for all flows, except the flow originating at *B*, where it is 10, for a targeted R=0.99999.
- The number of iterations of MOpt never exceeds h+1 in all the cases evaluated.

### 5.2. Load-Based Scheduling

**Figure 5:**
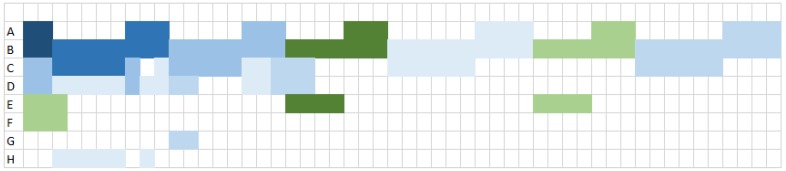
*Load-based* schedule with MFair.

**Figure 6:**
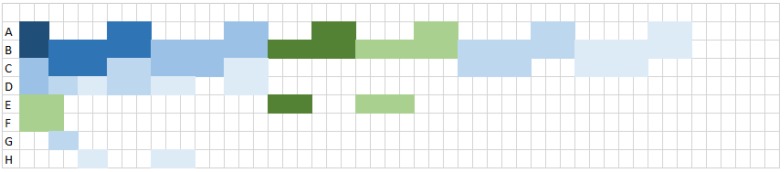
*Load-based* schedule with MOpt.

Let us see how these transmissions are scheduled, assuming a per-flow approach and more precisely the selection of the *Load-based* scheduler, which schedules first the flow originating from the most loaded node. We recall that the load of a node is computed as the number of cells needed to transmit its own flows, plus the number of cells needed to receive and transmit the flows originating from its descendants. Figure 5 and Figure 6 depict the *Load-based* schedule of the seven flows generated by sensor nodes with MFair and MOpt, respectively, assuming a targeted end-to-end reliability of 0.9. In both cases, the *Load-based* scheduler schedules the flows in the same order, starting with the most loaded node *B*, the scheduling order is B,C,D,E,H,F,G, although Load(B)=52 cells with MFair and only 46 with MOpt. The two resulting schedules are optimal in terms of slots needed because node *B*, the most loaded node, is kept busy in all slots of both schedules. However, MFair requires exactly 52 slots to schedule the 72 transmissions, (see Figure 5), whereas MOpt requires only 46 slots to schedule the 64 transmissions, (see Figure 6). In this simple configuration with seven flows, MOpt allows for saving eight transmissions, which represents an improvement of 11% in the number of transmissions to schedule. In addition, MOpt allows for saving six slots, which reduces by 11.5% the number of slots used.

In the *Load-based* schedule obtained with MFair and depicted in Figure 5, we have MinSize=52 slots. Hence, for a slot duration of 7.25 ms, the smallest maximum latency that can be achieved with MFair is equal to (52+51)*7.25=0.7465 s. The average energy consumption per slotframe of node *B*, the greatest loaded node, is equal to: (22×TXCharge+30×RXCharge)/SFDuration, where SFDuration denotes the slotframe duration. With an initial energy of 2821.5 mAh, the default slotframe size of 101 slots and a slot duration of 7.25 ms, this node will have a lifetime of 39 days and a maximum latency of $\left(101-1+52\right)*7.25*10^{-3}=1.102$ s. To meet a lifetime of one year, the slotframe size should be greater than or equal to 933 slots, with a maximum latency of $\left(933-1+52\right)*7.25*10^{-3}=7.0905$ s.

With the *Load-based* schedule obtained with MOpt and depicted in Figure 6, we have MinSize=46 slots. Hence, for a slot duration of 7.25 ms, the smallest maximum latency that can be achieved with MOpt is equal to (46+45)*7.25=0.65975 s, which represents an improvement of 13.67%. It becomes 1.0585 s for the default slotframe size of 101 slots. The average energy consumption of *B* per slotframe becomes (20×TXCharge+26×RXCharge)/SFDuration, leading to a network lifetime of 44 days for the default slotframe size of 101 slots and a maximum end-to-end latency of (101−1+46)*7.25=1.0585 s. To meet a lifetime of one year, the slotframe size should be greater than or equal to 830 slots, with a maximum latency of $\left(830-1+46\right)*7.25*10^{-3}=6.34375$ s, which represents an improvement of 12.38% with regard to MFair. For a slotframe size of 933 slots, MOpt would provide a maximum latency of 7.0905 s, a decrease of 11.77% with regard to MFair and a network lifetime of 410 days instead of 365 for MFair, an increase of 12.40%.

Table 8 points out the trade-off between the maximum end-to-end latency and network lifetime by listing the results obtained by MFair and MOpt for different slotframe sizes: 52, 101 and 933 slots. To increase the network lifetime by increasing the slotframe size, provided that the application still generates the same number of messages per slotframe, leads to an increase in maximum latency. MOpt provides a shorter maximum end-to-end latency because of a smaller schedule size (i.e., smaller number of slots used).

## 6. Performance Results of a TSCH Network with 50 Nodes

For the performance evaluation of a TSCH network with 50 nodes, we use the 6TiSCH simulator [28], which has been designed for a fast prototyping. In [8], network performances are evaluated on two specific applications running on a same network. In this particular configuration, randomly deployed sensors are only in charge of generating messages that they forward to a close relay, whereas relays are deployed according to a triangular grid. Since a more generic configuration is representative of much more applications, we focus on a generic data gathering application running on random network topologies.

### 6.1. Simulation Parameters

The network topology is a random topology such that any mote has at least three neighbors (i.e., three motes with which PDR≥0.5). For each wireless link, the PDR value is computed according to the Pister–Hack model [29]. The 6TiSCH protocol stack is used with RPL as the routing protocol with the ETX metric and the Load-based scheduler as the scheduling function. RPL, MFair and MOpt use the PDR values computed for the wireless links considered. For each wireless link *i*, RPL deduces ETX(i) from PDR(i). The simulation parameters used to evaluate the KPIs are those given in Table 9. The deepest routing tree observed in the simulations is 7-hop deep, the shallowest is 4-hop deep, with a median of 5-hop.

Notice that, in the simulations done with the 6TiSCH stack, MaxTrans, the maximum number of transmissions of any message on any link is fixed, as in the standardized MAC TSCH protocol. In the 6TiSCH simulations, its value is set to 6. If after six transmissions the acknowledgment is not received, the message is discarded. This behavior has a strong impact on the latency. Since Assumption 4 is not met, the theoretical bound for the maximum latency given in Equation (6) is no longer valid, we use the new theoretical bound given in Section 4.4.

A legitimate question is why a schedule provides a number of Transmission cells (TX) for any given flow greater than MaxTrans, since a message that has not been acknowledged after MaxTrans transmissions is discarded. The justification is provided by the decrease in the average end-to-end latency and the maximum end-to-end latency as we will see in Section 6.3. This decrease is due to a greater number of opportunities to transmit.

### 6.2. End-To-End Delivery Rate

**Figure 7:**
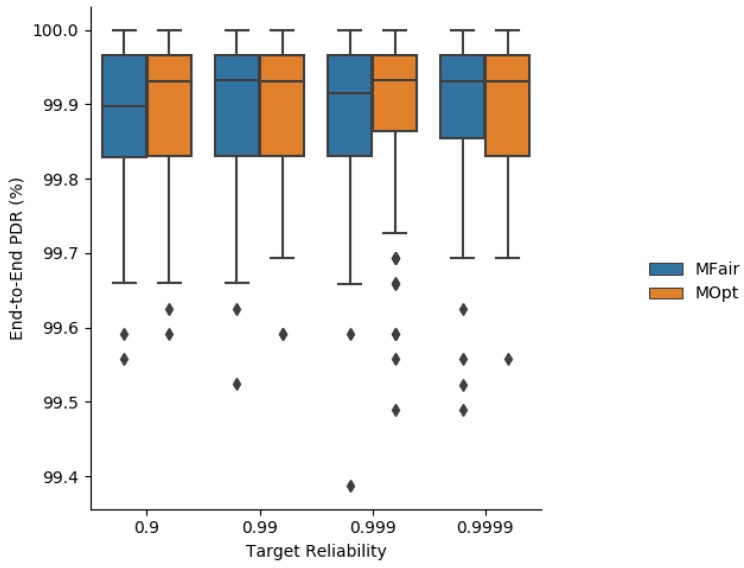
End-to-end delivery rate obtained with MFair and MOpt.

Simulation results about the average end-to-end delivery rate are depicted in Figure 7.

As expected, the end-to-end reliability is better with MOpt than with MFair because MOpt maximizes the end-to-end reliability provided for a minimum total number of transmissions per message.

### 6.3. End-To-End Latency

**Figure 8:**
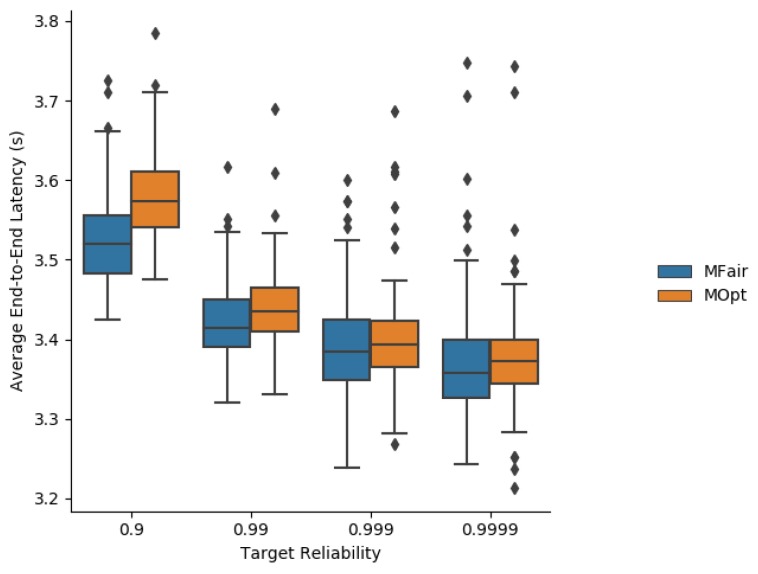
Average end-to-end latency obtained with MFair and MOpt.

Simulation results about the average end-to-end latency are depicted in Figure 8.

As MFair tends to compute a greater number of transmissions than MOpt, the schedule for MFair includes a greater number of cells in a slotframe whose size is kept identical for MOpt and MFair. The more cells, the more chance to send or forward. Since an application packet is generated at a random time point in the slotframe, the more transmission (TX) cells the node has, and the more chance to send the packet immediately. This is why the average end-to-end latency is shorter with MFair.

**Figure 9:**
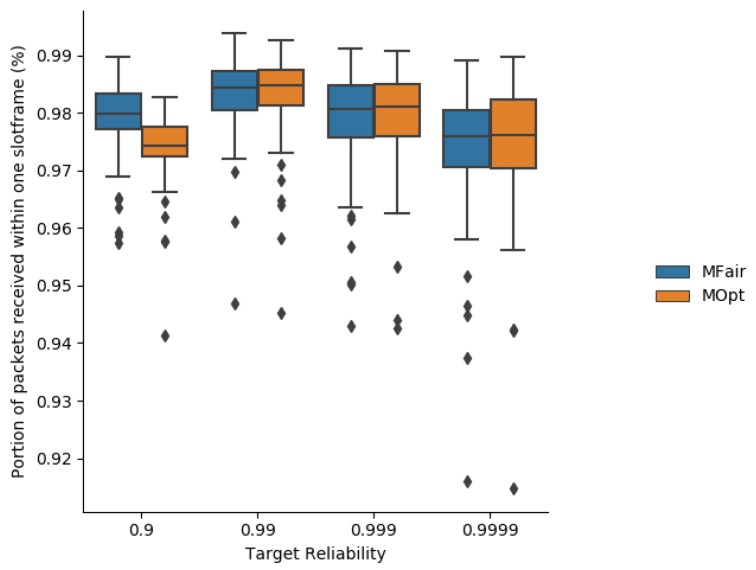
Percentage of packets delivered in one slotframe with MFair and MOpt.

Figure 9 shows the percentage of messages delivered in a single slotframe. It is smaller with MOpt than with MFair due to the greater number of TX cells granted by MFair.

**Figure 10:**
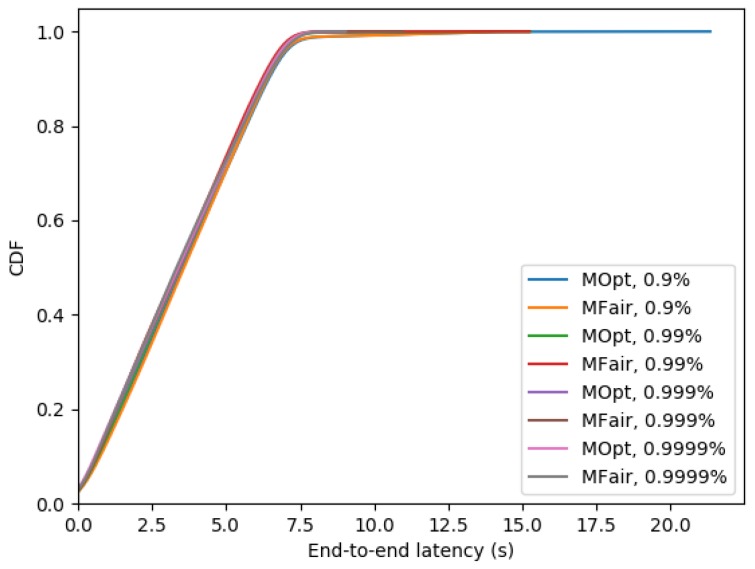
Cumulative distribution function of the end-to-end latency with MFair and MOpt.

Figure 10 depicts the cumulative distribution function of the end-to-end latency for MFair and MOpt. With MFair, 98% of messages reach the sink in one slotframe that is with an end-to-end latency ≤7 s. The gap between MFair and MOpt is smaller than 1%.

**Figure 11:**
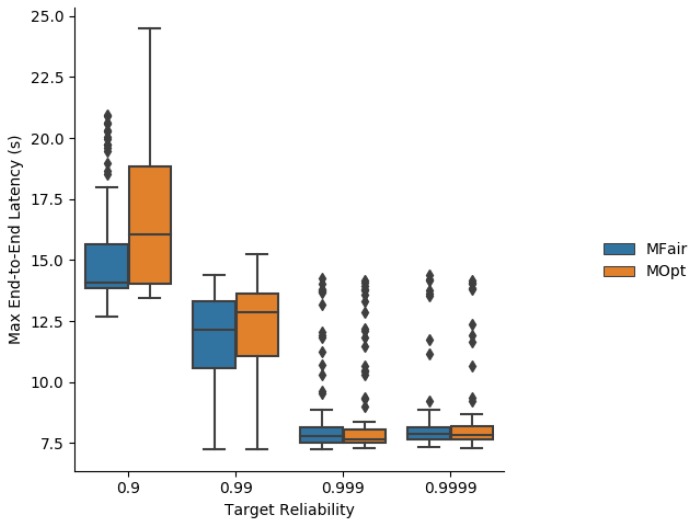
Maximum end-to-end latency obtained by simulation with MFair and MOpt.

The maximum end-to-end latency obtained by simulation with MFair and MOpt is illustrated in Figure 11. Since MFair allocates more TX cells and the slotframe size is kept identical for MFair and MOpt, MFair provides a shorter maximum end-to-end latency. For targeted reliabilities ≥0.999, MFair and MOpt provide very close maximum latencies.

**Figure 12:**
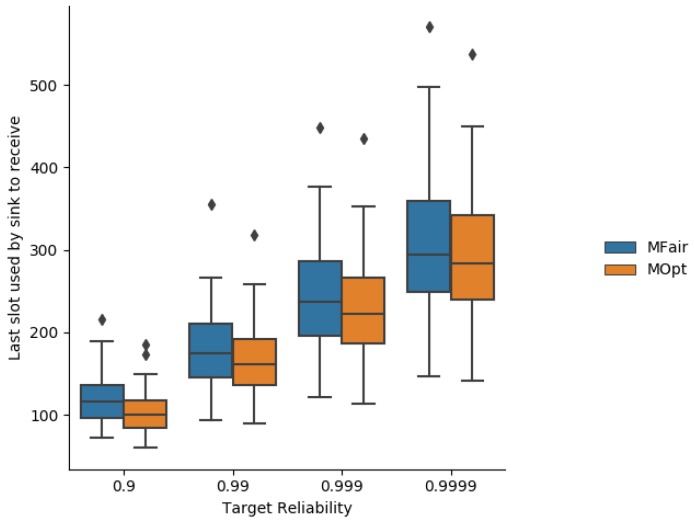
Maximum slot used by MFair and MOpt.

Figure 12 depicts the schedule size expressed as the number of slots used in the schedule of MFair and MOpt. Unsurprisingly, the schedule size increases with the targeted end-to-end reliability, due to a great number of transmissions on each link to reach the targeted end-to-end reliability. Whatever the targeted reliability, the schedule size is always shorter with MOpt than with MFair, as expected.

Table 10 compares the theoretical bound for the maximum end-to-end latency obtained by Equation (10) with the simulation results, for different values of the targeted end-to-end reliability and a given random topology. The maximum end-to-end latency obtained by simulation decreases when the targeted end-to-end reliability increases: a greater number of TX cells assigned to nodes give them more opportunities to transmit in a slotframe. With the theoretical bound, the decrease is obtained when the number of TX Cells assigned to a node increases to reach the targeted end-to-end reliability. Whatever the targeted reliability, the bound given by Equation (10) and the simulation results are not close. The theoretical bound could be refined to take more information into account.

Table 10 also provides the end-to-end reliability. As expected, MOpt provides an end-to-end reliability better than MFair. However, for a targeted end-to-end reliability ≥0.999, MFair fails to achieve the requested reliability, when MaxTrans is left fixed to 6 instead of being dynamically tuned according to the values computed by MFair or MOpt.

### 6.4. Duty Cycle

With regard to network lifetime and energy consumption, we consider the busiest node excluding the root (sink) node, since it is supposed to be mains-powered. This busiest node determines the network lifetime. The duty cycle on this busiest node is computed as:

(11) $$Duty\_Cycle=\frac{number\_of\_slots\_used\_on\_the\_busiest\_node}{schedule\_size\_in\_slots}.$$

Note that Equation (11) is meant for the following comparison, which does not represent actual radio duty cycle. In TSCH, a device does not turn on its radio all the time even during an active slot.

**Figure 13:**
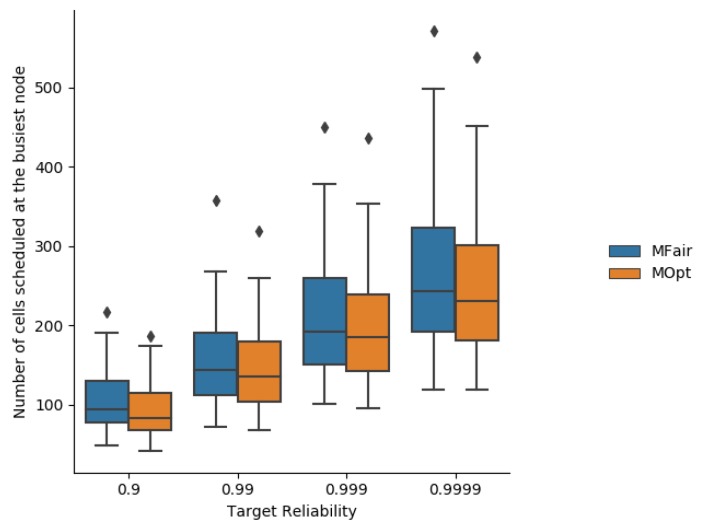
Number of cells assigned by MFair and MOpt on the busiest node.

Figure 13 depicts the number of cells assigned to the busiest node in one simulation run for each targeted end-to-end reliability.

Table 11 gives the duty cycle on the busiest node with MFair and MOpt. This gives an insight on network lifetime which is determined by the lifetime of the busiest node.

As a consequence, for high targeted reliabilities (i.e., ≥0.999), MFair and Mopt give close end-to-end latencies at a greater energy cost for MFair.

### 6.5. Impact of MaxTrans, a TSCH Parameter

We now study the impact of MaxTrans the maximum number of transmissions of a message in TSCH, whose default value is 6. We set MaxTrans to the value of 20, which is greater than the maximum number of transmissions on each link computed by MFair or MOpt. We run the same simulations as previously and evaluate the impact on the end-to-end reliability and the end-to-end latency.

**Figure 14:**
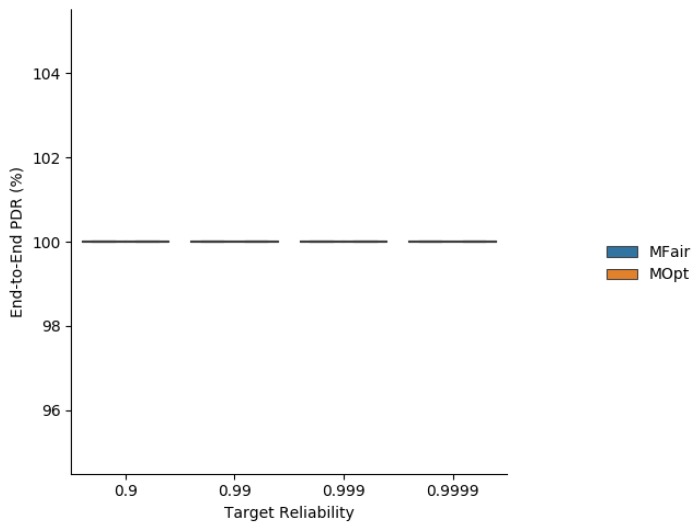
End-to-end delivery rate obtained with MFair and MOpt for MaxTrans=20 transmissions.

As expected, the end-to-end reliability depicted in Figure 14 is increased both with MOpt and MFair, when the maximum number of transmissions per message in TSCH is set to 20. It is very close to 100%, for all the values of the targeted reliability tested. Notice, however, that increasing MaxTrans may lead to a network overload resulting in violations of the maximum acceptable latency *L*. To avoid that, messages whose lifetime is greater than or equal to *L* should not be transmitted and should be discarded.

**Figure 15:**
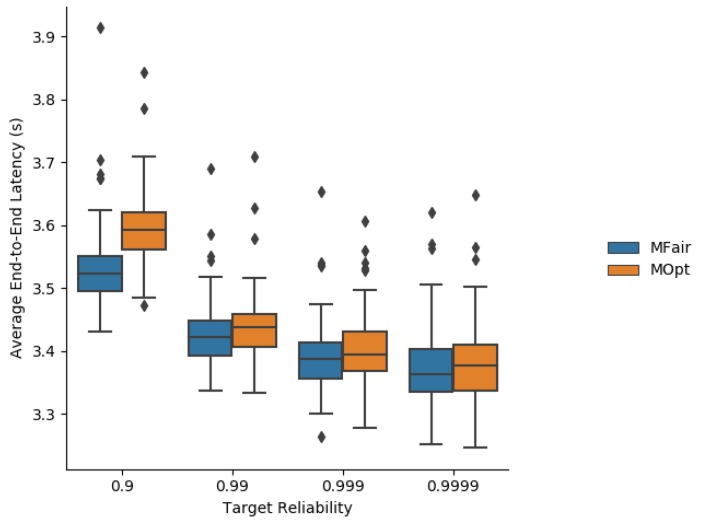
Average end-to-end latency obtained with MFair and MOpt for MaxTrans = 20 transmissions.

The consequence of increasing MaxTrans to 20 is an increase in the average latency for both MFair and MOpt, as shown in Figure 15. The shortest average latency is still provided by MFair. However, the gap decreases for high targeted end-to-end reliabilities (i.e., ≥0.999). Using a value of MaxTrans greater than the value required by MFair or MOpt on a given link may result in an increase in end-to-end latency and energy consumption for a very strongly limited gain in end-to-end reliability.

## 7. Comparison with Kausa

Since Kausa [8] is a well-known centralized scheduler that takes into account the unreliability of wireless links and adopts a per-flow approach to meet KPIs, we now compare MOpt to Kausa when run in a 6TiSCH network.

### 7.1. Our Kausa Implementation in the 6TiSCH Simulator

In the 6TiSCH simulator, as in any 6TiSCH implementation compliant with the standard, routing is done by the RPL protocol with the ETX metric. We simulated Kausa on the 6TiSCH simulator and ran it with the generic configuration defined in Section 6.1. We recall that the value of MaxTrans is fixed to 6. In these conditions, the main differences between Kausa and our approach are:

- Kausa selects first the flow requesting the highest end-to-end reliability, then the shortest end-to-end latency and finally the flow originating from the farthest node of the sink. Our approach selects the flow originating from the most loaded sensor node (i.e., the sensor node needing the largest number of Tx+Rx cells).
- For any flow *f*, Kausa starts by assigning cells to the most loaded node visited by *f*. Then, Kausa goes backward to the source of *f*. Finally, Kausa goes upward from the most loaded node to the sink. In our approach, cells are assigned to nodes visited by *f* in a cascading way from the source of *f* up to the sink. It follows that our approach is easier to implement.
- For any flow *f*, Kausa minimizes the number of retransmissions on the most loaded node, whereas we minimize the total number of retransmissions on the path of *f*.

Notice that we implement an optimization of Kausa for 6TiSCH, enabling any node to use its next Tx cell to transmit any message to its parent, as done in our approach. This is not true in the published version of Kausa, where any node *N* is not allowed to use cells assigned to a flow *f* for another flow $f^{\prime }$ visiting *N*.

### 7.2. End-To-End Latency

**Figure 16:**
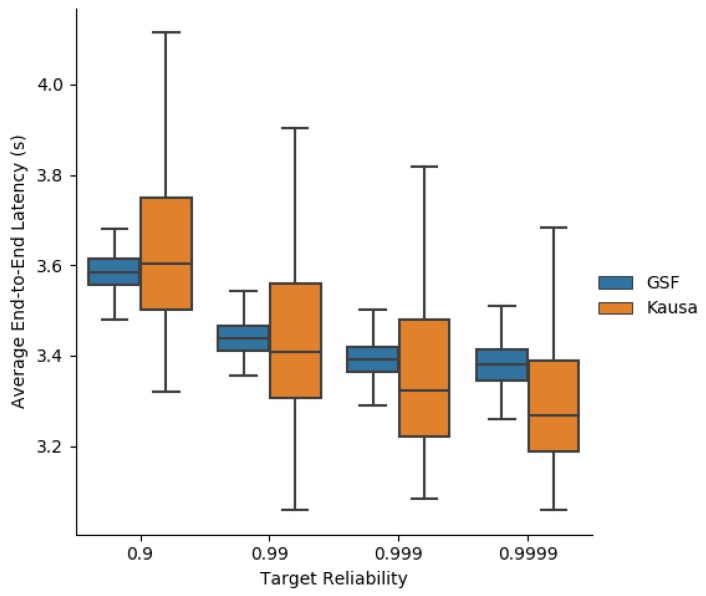
Average end-to-end latency obtained with MOpt and Kausa.

Figure 16 depicts the average end-to-end latency obtained by MOpt and Kausa. They both provide close values. However, MOpt provides a much smaller variance of the average end-to-end latency than Kausa, making it more predictable, even for high targeted reliabilities.

**Figure 17:**
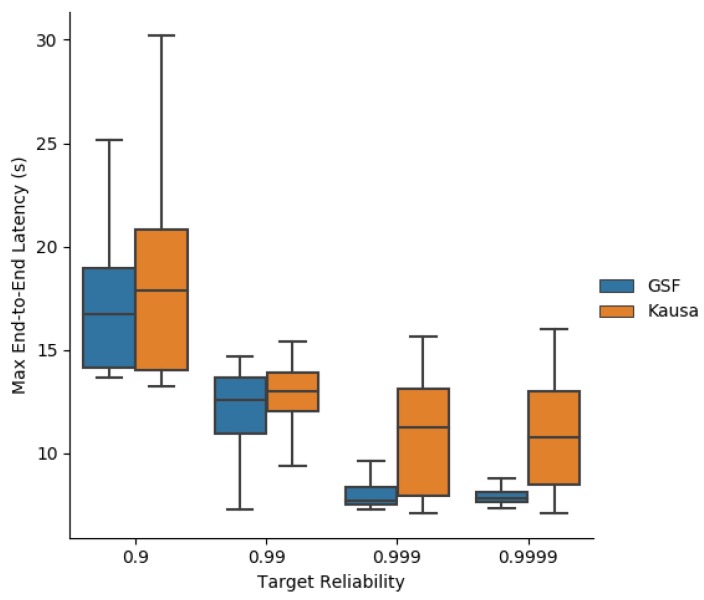
Maximum end-to-end latency obtained with MOpt and Kausa.

The same conclusion applies to the maximum end-to-end latency depicted in Figure 17. The predictability of performance is a property sought by industrial applications.

### 7.3. End-To-End Reliability

**Figure 18:**
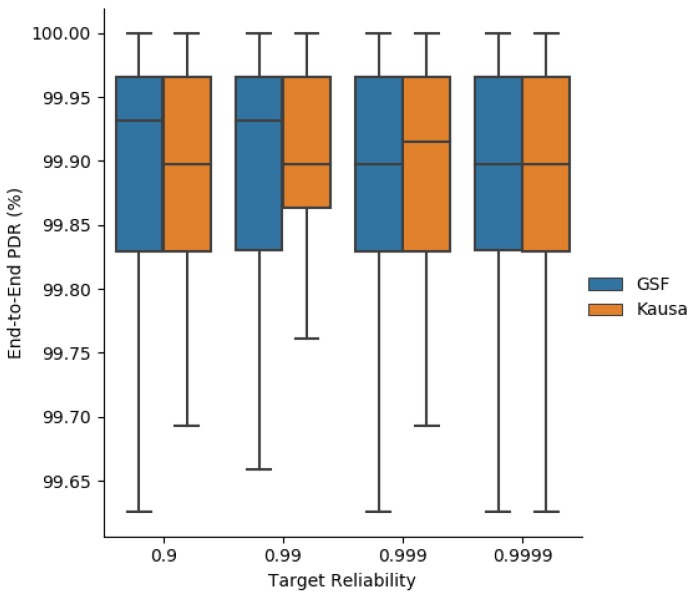
End-to-end delivery rate obtained with MOpt and Kausa.

With regard to the end-to-end PDR, we observe in Figure 18 that both MOpt and Kausa ensure a PDR greater than the targeted one when it belongs to the interval [0.9, 0.999]. For greater values, the PDR is not met because the value of MaxTrans=6 is too small to reach the targeted end-to-end reliability. In addition, MOpt tends to provide a greater median value than Kausa.

### 7.4. Duty Cycle

**Figure 19:**
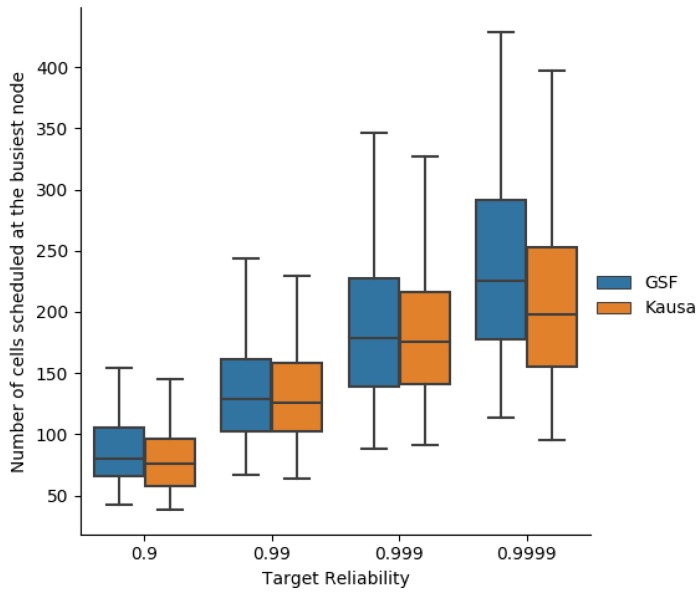
Number of cells used at the busiest node with MOpt and Kausa.

Since the energy consumption can be deduced from the duty cycle of the busiest node, we compare the number of cells scheduled at the busiest node by Kausa and MOpt, as depicted in Figure 19. Both provide close values for a targeted end-to-end reliability less than or equal to 0.999. For a higher reliability, Kausa provides a smaller number of cells due to a number of transmissions scheduled per message less than or equal to MaxTrans, whereas MOpt may schedule a larger number, explaining this result.

### 7.5. Schedule Size

**Figure 20:**
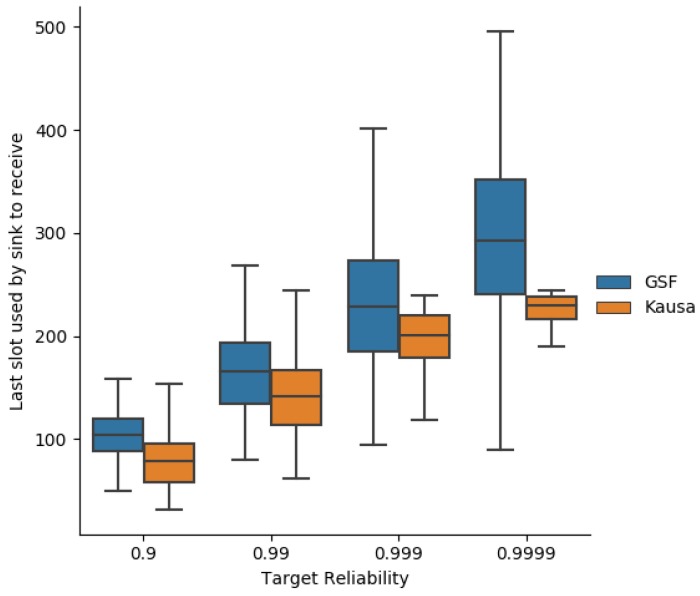
Schedule size obtained with MOpt and Kausa.

The schedule size, illustrated in Figure 20, is a little greater with MOpt than with Kausa. As a consequence, a node has fewer opportunities to transmit with Kausa than with MOpt, leading to a higher and less predictable end-to-end latency.

## 8. Conclusions

TSCH is a very promising technology for the IoT. It is now necessary to evaluate the performances it can provide to IoT applications. These performances are evaluated by means of three KPIs: maximum end-to-end latency *L*, end-to-end reliability *R* and network lifetime *T*. This IoT network has to guarantee that at least R% of messages generated by sensors are delivered to the sink with a latency less than or equal to *L*, while the network lifetime is at least *T*. In this paper, we have proposed two methods MFair and MOpt to achieve a targeted end-to-end reliability taking into account the unreliability of wireless links. The trade-offs between end-to-end latency, network lifetime and end-to-end reliability have been pointed out. In addition, we have shown that minimizing the total number of transmissions of a message to reach the sink with MOpt saves 12% of network lifetime in a small network of eight nodes, assuming a *Load-based* schedule of flows. As expected, MOpt provides a better end-to-end reliability and a longer network lifetime than MFair. However, the average end-to-end latency provided by MFair is smaller. These results scale up to a 50-node network representative of real deployments, as shown by simulation results obtained with the 6TiSCH simulator. However, using a fixed maximum number of transmissions MaxTrans equal to the default value of the TSCH protocol, instead of using the value computed by MFair or MOpt for the link and the flow considered, may lead to a violation of the targeted end-to-end reliability: messages that have not been acknowledged after MaxTrans transmissions are discarded. Compared to Kausa, a KPI-aware, state-of-the-art scheduler, our approach is simpler to implement and ensures more predictable end-to-end performances, which is an essential property for industrial applications.

## Figures and Tables

**Table 1:** Main notations.

Name	Meaning
*R*	the targeted end-to-end reliability
*L*	the targeted end-to-end latency
*T*	the targeted network lifetime
*j*	the link from node $N_{j}$ to its parent in the routing tree
$M_{j}$	the maximum number of transmissions of any message transmitted on link *j*
$P_{j}$	the probability of successful acknowledgment receipt after a single message transmission on link *j*
$R_{j}$	the probability of successful acknowledgment receipt on link *j* after a maximum number $M_{j}$ of transmissions

**Table 2:** Parameters used for network lifetime computation.

Parameter	Value
Initial energy of node powered by 2 Energizers L-91 AA batteries	2821.5 mAh
Transmit a data frame & receive its acknowledgment	54.5 μC
Receive a data frame & transmit its acknowledgment	32.6 μC
IdleListen	6.4 μC
Sleep	0 μC

**Table 3:** Comparison of MFair and MOpt for a targeted end-to-end reliability R = 0.9 on seven flows.

		Maximum Number of Transmissions for Targeted R = 0.9										Total Number of Transmissions per msg		End-To-End Reliability	
Flow	Hop	P = 0.7		P = 0.5		P = 0.8		P = 0.6		P = 0.9					
		**Fair**	**Opt.**	**Fair**	**Opt.**	**Fair**	**Opt.**	**Fair**	**Opt.**	**Fair**	**Opt.**	**Fair**	**Opt.**	**Fair**	**Opt.**
B	1	2	2									2	2	0.91	0.91
C	2	3	3	**5**	**4**							**8**	**7**	0.9425	0.91218
E	2	3	3					**4**	**3**			**7**	**6**	0.9480	0.9107
D	3	3	3	5	5	**3**	**2**					**11**	**10**	0.9350	0.90489
F	3	3	3					4	4			10	10	0.92249	0.92249
G	4	**4**	**3**	**6**	**5**	3	3			2	2	**15**	**13**	0.95890	0.92570
H	4	**4**	**3**	**6**	**5**	3	3					**19**	**16**	0.95345	0.90583

**Table 4:** Comparison of MFair and MOpt for a targeted end-to-end reliability R = 0.99 on seven flows.

		Maximum Number of Transmissions for Targeted R = 0.99										Total Number of Transmissions per msg		End-To-End Reliability	
Flow	Hop	P = 0.7		P = 0.5		P = 0.8		P = 0.6		P = 0.9					
		**Fair**	**Opt.**	**Fair**	**Opt.**	**Fair**	**Opt.**	**Fair**	**Opt.**	**Fair**	**Opt.**	**Fair**	**Opt.**	**Fair**	**Opt.**
B	1	4	4									4	4	0.9919	0.9919
C	2	5	5	8	8							13	13	0.993673	0.993673
E	2	5	5					6	6			11	11	0.99348	0.99348
D	3	5	5	**9**	**8**	4	4					**18**	**17**	0.99402	0.99208
F	3	5	5	5				**7**	**6**			**17**	**16**	0.9935	0.99106
G	4	5	5	**9**	**8**	4	4			3	3	**21**	**20**	0.99303	0.99109
H	4	5	5	9	**9 8**	3	3					**27**	**26**	0.99208	0.99014

**Table 5:** Comparison of MFair and MOpt for a targeted end-to-end reliability R = 0.999 on seven flows.

		Maximum Number of Transmissions for Targeted R = 0.99										Total Number of Transmissions per msg		End-To-End Reliability	
Flow	Hop	P = 0.7		P = 0.5		P = 0.8		P = 0.6		P = 0.9					
		**Fair**	**Opt.**	**Fair**	**Opt.**	**Fair**	**Opt.**	**Fair**	**Opt.**	**Fair**	**Opt.**	**Fair**	**Opt.**	**Fair**	**Opt.**
B	1	6	6									6	6	0.99927	0.99927
C	2	7	7	11	11							18	18	0.99929	0.99929
E	2	7	7					**9**	**8**			**16**	**15**	0.99951	0.99912
D	3	7	7	**12**	**11**	**5**	**6**					24	24	**0.99921**	**0.999229**
F	3	7	7					9	9			23	23	0.99930	0.99930
G	4	7	8	**12**	**11**	6	6			4	4	**29**	**28**	0.99937	0.99922
H	4	7	7	12	12	6	6					37	37	0.999229	0.999229

**Table 6:** Comparison of MFair and MOpt for a targeted end-to-end reliability R = 0.9999 on seven flows.

		Maximum Number of Transmissions for Targeted R = 0.99										Total Number of Transmissions per msg		End-To-End Reliability	
Flow	Hop	P = 0.7		P = 0.5		P = 0.8		P = 0.6		P = 0.9					
		**Fair**	**Opt.**	**Fair**	**Opt.**	**Fair**	**Opt.**	**Fair**	**Opt.**	**Fair**	**Opt.**	**Fair**	**Opt.**	**Fair**	**Opt.**
B	1	8	8									8	8	0.999934	0.999934
C	2	9	9	15	15							24	24	0.9999498	0.9999498
E	2	9	9					11	11			20	20	0.99993837	0.99993837
D	3	9	9	**15**	**14**	7	7					**31**	**30**	0.999937	0.999918
F	3	9	9					**12**	**11**			**30**	**29**	0.99994386	0.999906
G	4	9	9	**16**	**15**	7	7			5	5	**37**	**36**	0.999942	0.999927
H	4	9	9	**16**	**15**	7	7					**48**	**46**	0.999937	0.999906

**Table 7:** Comparison of MFair and MOpt for a targeted end-to-end reliability R = 0.99999 on seven flows.

		Maximum Number of Transmissions for Targeted R = 0.99										Total Number of Transmissions per msg		End-To-End Reliability	
Flow	Hop	P = 0.7		P = 0.5		P = 0.8		P = 0.6		P = 0.9					
		**Fair**	**Opt.**	**Fair**	**Opt.**	**Fair**	**Opt.**	**Fair**	**Opt.**	**Fair**	**Opt.**	**Fair**	**Opt.**	**Fair**	**Opt.**
B	1	10	10									10	10	0.9999941	0.9999941
C	2	11	11	**18**	**17**							**29**	**28**	0.99999441	0.9999906
E	2	11	11					**14**	**13**			**25**	**24**	0.99999554	0.99999152
D	3	11	11	**19**	**18**	8	8					**38**	**37**	0.99999376	0.99999185
F	3	11	11					14	14			36	36	0.99999377	0.99999377
G	4	11	11	**19**	**18**	9	9			6	6	**45**	**44**	0.9999948	0.9999929
H	4	11	11	**19**	**18**	9	9					**58**	**56**	0.9999939	0.9999909

**Table 8:** The trade-off of end-to-end latency versus network lifetime for MFair and MOpt.

	MFair			MOpt			Relative Improvement		
	Slotframe(slots)			Slotframe(slots)			Slotframe(slots)		
	**52**	**101**	**933**	**52**	**101**	**933**	**52**	**101**	**933**
Max. latency (s)	0.74675	1.102	7.134	0.70325	1.0585	7.0905	5.82%	3.94%	0.61%
Lifetime (days)	20.35	39.54	365.28	22.87	44.42	410.41	10.98%	12.35%	12.35%

**Table 9:** Simulation parameters.

	Parameter	Value
Config.	Number of nodes	50
	Number of Channels	16
	Topology	random
	Link reliability	computed
TSCH	Slot duration	10 ms
	Slotframe size	700 slots
	Secure join	disabled
	Keep-alive (L2)	disabled
	Transmit queue size	10 packets
	Max of retransmissions	5 (i.e., max of 6 transmissions)
Routing	RPL	with ETX metric where DAO is disabled
	a stable routing topology	after 60 min
Scheduling	Scheduling function	Load-based scheduler
Application	is run & measures made	during the next 60 min
	packet generation interval on each sensor node	[57 s, 63 s]
Simulation	100 runs per pair (algorithm, targeted reliability)	algorithm ∈{MOpt,MFair}
		target. reliability ∈{0.9,0.99,0.999,0.9999}

**Table 10:** Comparison of the theoretical maximum end-to-end latency and the simulation results for MFair and MOpt, taken from particular simulations having the same routing topology.

			Targeted End-To-End Reliability			
			0.9	0.99	0.999	0.9999
Schedule size (slots)	MFair		96	154	199	249
	MOpt		84	134	190	246
	relative improvement		12.5%	12.98%	4.52%	1.20%
Min TX cells per (node, flow)	MFair		2	2	2	3
	MOpt		2	2	2	3
Maximum end-to-end latency (s)	MFair	theory	28.96	29.54	29.99	16.49
		simulation	13.79	13.93	9.64	7.70
	MOpt	theory	28.84	29.34	29.90	16.46
		simulation	19.93	13.94	7.43	7.69
End-to-end reliability	MFair		0.997959	0.998977	0.998978	0.999659
	MOpt		1	0.998294	0.999318	1

**Table 11:** Comparison of the duty cycle of the busiest node for MFair and MOpt.

		Targeted End-To-End Reliability			
		0.9	0.99	0.999	0.9999
Schedule size (slots)	MFair	96	154	199	249
	MOpt	84	134	190	246
Cells assigned to busiest node	MFair	77	103	127	159
	MOpt	63	87	122	149
Duty cycle of busiest node	MFair	11%	14.71%	18.14%	22.71%
	MOpt	9%	12.42%	17.42%	21.28%
Relative gain		18.18%	15.53%	3.93%	6.29%
